# Non-linear responses of net ecosystem productivity to gradient warming in a paddy field in Northeast China

**DOI:** 10.7717/peerj.9327

**Published:** 2020-06-10

**Authors:** Yulu Sun, Fuyao Qu, Xianjin Zhu, Bei Sun, Guojiao Wang, Hong Yin, Tao Wan, Xiaowen Song, Qian Chen

**Affiliations:** 1 College of Agronomy, Shenyang Agricultural University, Shenyang, Liaoning, China

**Keywords:** Carbon cycle, Climate change, Non-linear response, Terrestrial ecosystem, Warming

## Abstract

Global warming has a known impact on ecosystems but there is a lack of understanding about its impact on ecosystem processes. Net ecosystem productivity (NEP) and its components play a key part in the global carbon cycle. Analysing the impact of global warming on NEP will improve our understanding of how warming affects ecosystems. In our study, conducted in 2018, five warming treatments were manipulated (0 W, 500 W, 1000 W, 1500 W, and 3000 W) using three repetitions of far infrared open warming over a paddy field in Northeast China. NEP and its two related components, gross primary productivity (GPP) and ecosystem respiration (ER), were measured using the static chamber-infrared gas analyser method to explore the effects of different warming magnitudes on NEP. Results showed that measurement dates, warming treatments, and their interactions significantly affected NEP, ER, and GPP. Warming significantly increased NEP and its components but they showed a non-linear response to different warming magnitudes. The maximum increases in NEP and its components occurred at 1500 W warming. NEP is closely related to its components and the non-linear response of NEP may have primarily resulted from that of GPP. Gradient warming non-linearly increased GPP in the paddy field studied in Northeast China, resulting in the non-linear response of NEP. This study provides a basis for predicting the responses of carbon cycles in future climate events.

## Introduction

Global warming is a widely accepted and validated phenomenon (Ma et al., 2017). The latest World Meteorological Organization (WMO) statement (https://library.wmo.int/doc_num.php?explnum_id=5789) suggests that the global mean temperature for 2018 is 0.99 ± 0.13 °C higher than that of the preindustrial baseline (1850–1900) and was the fourth warmest on record. 2015 to 2018 were the four warmest years in the global temperature record (WMO, 2019). Understanding the impacts of warming on ecosystems may provide a theoretical basis for mitigating climate change, which is a pressing ecological issue (Fu, Niu & Zhao, 2005).

Temperature affects the growth, reproduction, and biomass distribution of plants. Temperature increases are shown to impact the community structure (Blumenthal et al., 2016; Xu et al., 2015; Yvon-Durocher et al., 2010), phenology (Wolkovich et al., 2012), and carbon cycle processes (Gao et al., 2010; Liu & Chen, 2000; Tokarska & Gillett, 2018) of plants. Net ecosystem productivity (NEP) and its components, including gross primary productivity (GPP) and ecosystem respiration (ER) play a key part in the global carbon cycle. Analysing the impacts of warming on NEP will help understand how warming affects the carbon cycle and its underlying mechanisms.

Studies of this type could be fulfilled through models and manipulating experiments. Manipulating experiments provide direct evidence of ecosystem responses to warming and are validated by models. Manipulating experiments have been conducted to investigate how warming affects NEP in the Qinghai-Tibetan Plateau meadow (Ganjurjav et al., 2015; Mu et al., 2017), the grasslands of the Great Plains of North America (Niu et al., 2013; Pendall et al., 2013), and the polar ecosystems of Northwest Greenland (Jones et al., 1998; Oberbauer et al., 2007). However, these studies only investigated whether increasing temperature affected NEP and its mechanisms via two warming treatments (warming and control) (Ganjurjav et al., 2015; Lupascu et al., 2013), while the predicted temperatures obviously varied under different climate scenarios (Fang et al., 2011). Some studies have investigated the effects of warming magnitudes on NEP with warming experiments (Fu, Niu & Dukes, 2015) but the native differences among ecosystems confounded the responses of NEP to the various temperature increases. Sharp et al. (2013) found a non-linear response of NEP to different warming magnitudes using a manipulating experiment in Northwest Greenland. The response of NEP to divergent warming magnitudes in other ecosystems was not well documented.

The paddy field is an important ecosystem in Northeast China, accounting for nearly one-fifth of its planting area. The simple, drought-free structure of the paddy field (flooded cultivation of a single-season paddy) is beneficial for fully understanding warming effects. The high carbon stock and temperature sensitivity of this area is useful to determine how future warming will affect this ecosystem.

We performed a gradient warming experiment using far infrared heat lamps in a paddy field in Northeast China. This study aimed to reveal the effects of gradient warming on NEP and its underlying mechanisms in this ecosystem. Our study may help to demonstrate the responses of terrestrial ecosystems to future climate change.

## Materials & Methods

### Study site

Our experiment was conducted from May to October 2018 in a paddy field (123°33′E, 41°49′N) at Shenyang Agricultural University. The experimental field is in a semi-arid and semi-humid monsoon climate with a mean annual temperature of 13.1 °C and a mean annual precipitation of 610 mm. North-japonica 2 seedlings were transplanted into the growing field on May 31, 2018, with a row space of 30 cm ×13 cm and 3 plants per pot. Fertilizer was applied to the field using a base fertilizer (96 kg N, 69 kg P_2_O_5_ and 45 kg K_2_O), a greening fertilizer (34.5 kg N), and a tiller fertilizer (34.5 kg N) every hectare. The applied water volume and weeding techniques were similar among treatments and were related to local practices.

### Experimental design

We used a far infrared open warming system (Dong et al., 2011; Nijs et al., 1997) consisting of a heating component, a power element, and control and monitoring parts. The heating component had a far infrared heated lamp, an iron bracket, and a white stainless steel reflector mounted 60–70 cm above the canopy that was manually lifted according to the rice height. Power was provided by an AC 220V power station. A computer served as the control part while the thermocouple thermometer monitored the real time air temperature around the canopy and below the lampshade.

The warming magnitude was set to increase approximately 1.5 °C, in accordance with the Paris Agreement to control the global mean temperature within that measurement. Different warming magnitudes were achieved using far infrared lamps with different powers, given that 1000 W warming per hour increases the temperature approximately 1 °C. The 5 warming gradients were: control (CK), W1 (500 W), W2 (1,000 W), W3 (1,500 W), and W4 (3,000 W). Each treatment was replicated three times in an area 2 m ×2 m randomly distributed in the field. The intervals among the replicates and treatments were 3 m and 4 m, respectively.

### Carbon flux measurements

From June to October 2018, NEP and ER were measured by the static chamber-infrared gas analyser method every 20 days on sunny days between 8 a.m. and 11 a.m. (Niu et al., 2013). Five measurements were taken during the growing season.

A square PVC frame 0.5 m ×0.5 m was inserted into the soil of each plot at a depth of approximately 15 cm. A transparent chamber (0.5 m ×0.5 m ×1.2 m) was buckled to the frame and two small electric fans were installed on either side. The buckled chamber was attached to an infrared gas analyser (CIRAS-3 Portable Photosynthesis System, PP Systems, USA) to measure the CO_2_ concentration. The CO_2_ concentration in the chamber was recorded at 2-second intervals for a 90-second period after the transparent chamber was run for 30 s, to avoid capturing the interference of human activities. NEP was calculated based on 45 continuously observed CO_2_ concentrations (*ρ*_c_) in the transparent chamber.

The chamber was lifted into the direction of the wind and covered with a black cloth once the NEP observations were completed. The above steps were repeated to measure the CO_2_ concentration in the chamber. The carbon exchange between the ecosystem and the atmosphere was considered to be the ER since the chamber was shielded from light.

### Data analysis

We used 45 continuous CO_2_ concentration (*ρ*_c_) observations to estimate NEP and ER by calculating the *ρ*_c_ change rate (*dρ*_c_ /*dt*) from a linear fitting:

NEP $= \frac{\mathrm{V }}{\mathrm{A}} \frac{d{\rho }_{c}}{dt}  $ E$\mathrm{R}= \frac{V}{A} \frac{d{\rho }_{c}}{dt} $

where NEP is the net ecosystem productivity, ER is the ecosystem respiration, V is the chamber volume, A is the chamber covered area, *ρ*_c_ is the CO_2_ mass concentration, and *t* is time.

Gross primary productivity (GPP) was calculated as the algebraic sum of NEP and ER.

The repeated-measures analysis of variance (ANOVA) was used to assess the effects of the warming treatments on NEP, ER, and GPP. The intrinsic effect test and the inter-subject effect test were conducted to investigate the influences of measuring dates and warming treatments on NEP, ER, and GPP, respectively. Linear regression was employed to examine the responses of NEP and its components to temperature, and relationships between NEP and its components, whose performance was validated by both *p* value and R^2^. All statistical analyses were conducted with SPSS software (SPSS 22.0 for Windows, SPSS Inc., Chicago, IL, USA).

## Results

### Effects of warming on NEP

The measuring dates, warming treatments, and their interactions were all found to significantly affect NEP (Table 1, *p* < 0.01).

**Table 1:** Effects of measuring dates (T), warming treatments (W), and their interactions (T ×W) on net ecosystem production (NEP), ecosystem respiration (ER) and gross primary productivity (GPP) from RMANOVA.

Source	NEP			ER			GPP		
	DF	*F*	*P*	DF	*F*	*P*	DF	*F*	*P*
T	2.35	1,533.57	<0.01	1.76	3,287.11	<0.01	4.00	3,173.30	<0.01
W	4.00	459.76	<0.01	4.00	248.19	<0.01	4.00	487.55	<0.01
T × W	9.40	7.28	<0.01	7.03	38.90	<0.01	16.00	14.76	<0.01

**Figure 1:**
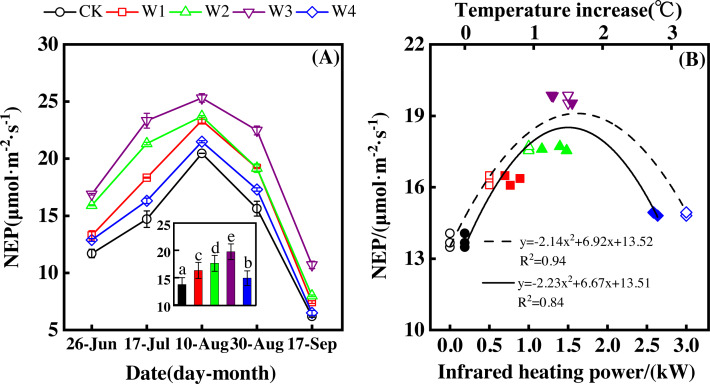
Responses of net ecosystem productivity (NEP) to measuring dates (A) and warming treatments (B) during the growing season. The warming treatments were CK (no warming), W1 (500 W warming), W2 (1,000 W warming), W3 (1,500 W warming), and W4 (3,000 W warming). The hollow symbols and dotted line were the responses to temperature, while the solid symbols and line were the responses to the warming treatments.

NEP showed a convex parabolic curve with a significant difference between measuring dates (Fig. 1A) during the growing season. From late June to early August, NEP monotonically increased and reached its peak (20.47–25.32 µmol m^−2^ s^−1^) in early August. However, NEP decreased from early August and reached its lowest value (6.19–10.70 µmol m^−2^ s^−1^) in the middle of September.

NEP significantly differed among treatments (Fig. 1A, *p* < 0.05). All warming treatments had a significantly higher NEP than the control (CK). In addition, different warming magnitudes resulted in significant differences in NEP. The highest NEP appeared at 1500 W warming (W3), corresponding to a temperature increase of 1.37 °C, with an NEP increase of 6.01 µmol m^−2^ s^−1^. Lower warming magnitudes both had an NEP increase, though the NEP increasing magnitudes were lower than that at 1500 W warming (W3). 500 W warming (W1) and 1000 W warming (W2) only led to an NEP increase of 2.58 and 3.89 µmol m^−2^ s^−1^, respectively. The minimum increase appeared at 3000 W warming (W4) with an NEP increase of 1.16 µmol m^−2^ s^−1^.

**Figure 2:**
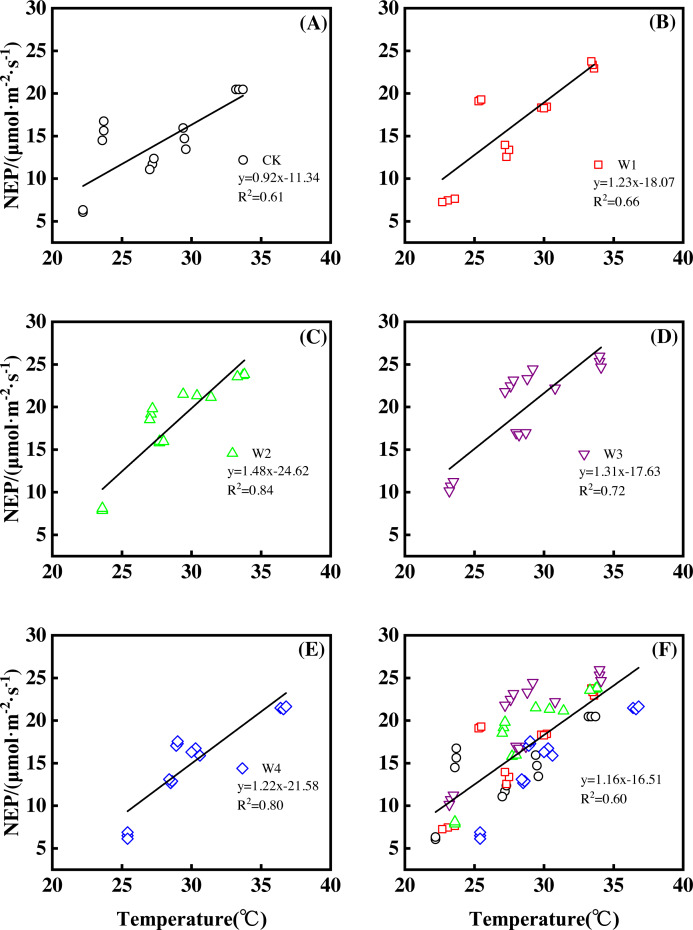
Responses of net ecosystem productivity (NEP) to temperature under different warming treatments (A–E) and under the combined effects (F) during the growing season. A–E were the responses of NEP to temperature under CK (no warming, (A), W1 (500 W warming, (B), W2 (1,000 W warming, (C), W3 (1,500 W warming, (D), and W4 (3,000 W warming, (E), respectively, while f was the responses of NEP to temperature under the combined effects of measuring dates and warming treatments.

Furthermore, different warming treatments resulted in diverse NEP increase rates (Figs. 2A–2E) during the whole growing season. CK had the lowest rate increase (0.92µmol m^−2^ s^−1^ per 1 °C temperature increase, Fig. 2A) while W2 had the highest rate increase (1.48 µmol m^−2^ s^−1^ per 1 °C temperature increase, Fig. 2C). Other warming treatments had a similar increasing rate around 1.3µmol m^−2^ s^−1^ per 1 °C temperature increase (Figs. 2B, 2D, 2E).

Therefore, NEP responded non-linearly to the gradient warming (Fig. 1B). As temperatures increased, the NEP revealed a convex parabolic curve with its peak value appearing between 1500 W and 2000 W warming, while its peak increasing rate occurred around 1000 W warming.

The increasing temperature led to a linear increase of NEP (Fig. 2F) with the combined effects of measurement dates and warming treatments. A 1 °C temperature increase resulted in an NEP increase of 1.16 µmol m^−2^ s^−1^ with an R^2^ of 0.60.

### Effects of warming on ER

The measuring dates, warming treatments, and their interactions also significantly impacted ER (Table 1, *p* < 0.01).

**Figure 3:**
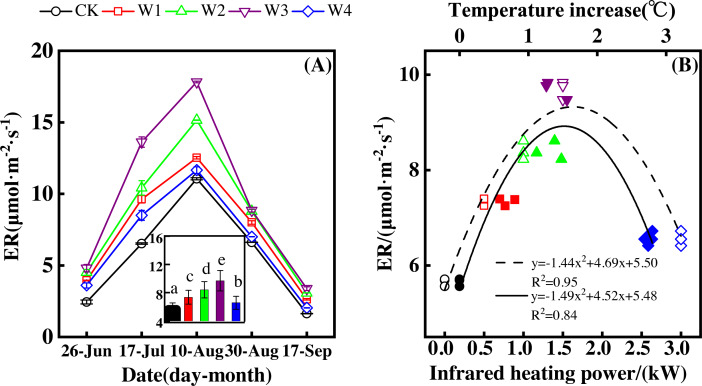
Responses of ecosystem respiration (ER) to measuring dates (A) and warming treatments (B) during the growing season. The warming treatments were CK (no warming), W1 (500 W warming), W2 (1,000 W warming), W3 (1,500 W warming), and W4 (3,000 W warming). The hollow symbols and dotted line were the responses to temperature, while the solid symbols and line were the responses to the warming treatments.

ER showed a convex parabolic curve over time during the growing season and differed significantly over the dates of measurement (Fig. 3A). From late June to early August, ER showed an upward trend and reached its peak (11.05–17.82 µmol m^−2^ s^−1^) in early August. ER started to decrease after early August and reached its lowest value at the end of the growing season.

ER differed significantly among treatments (Fig. 3A, *p* < 0.05). All warming treatments had a higher ER than the control (CK), which had the lowest ER of 1.63–3.38 µmol m^−2^ s^−1^. The highest ER appeared at 1,500 W warming (W3), which corresponded with a temperature increase of 1.37 °C, with an increase of 4.04 µmol m^−2^ s^−1^. 500 warming (W1) and 1,000 W warming (W2) resulted in an ER increase of 1.69 µmol m^−2^ s^−1^ and 2.75 µmol m^−2^ s^−1^, respectively, which were both lower than that of W3. 3000 W warming led to the least ER increase of 0.91 µmol m^−2^ s^−1^.

**Figure 4:**
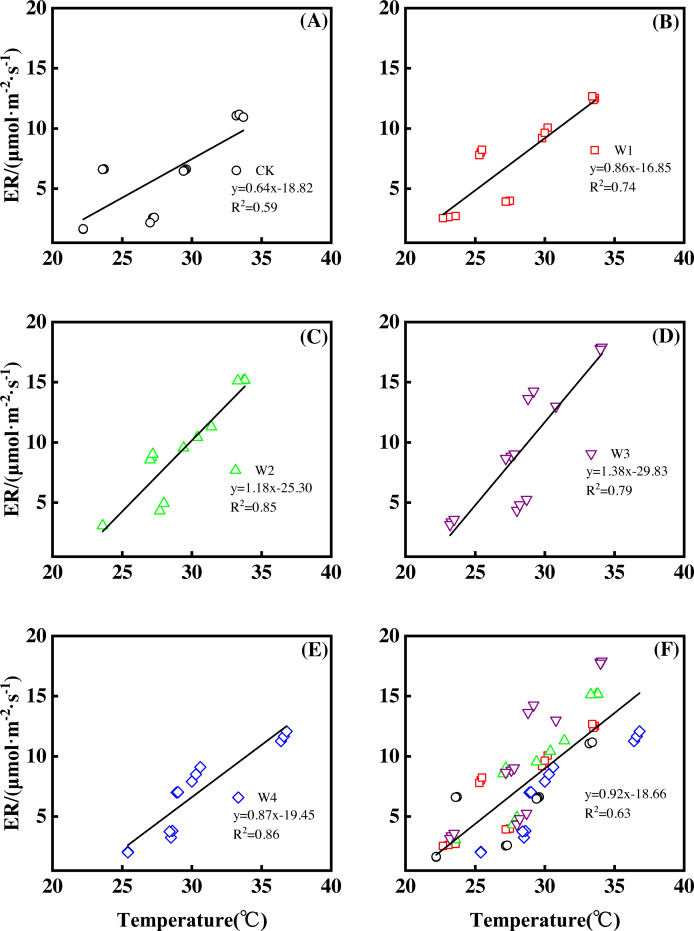
Responses of ecosystem respiration (ER) to temperature under different warming treatments (A–E) and under the combined effects (F) during the growing season. A–E were the responses of ER to temperature under CK (no warming, (A), W1 (500 W warming, b), W2 (1,000 W warming, (C), W3 (1,500 W warming, (D), and W4 (3,000 W warming, (E), respectively, while (F) was the responses of ER to temperature under the combined effects of measuring dates and warming treatments.

Different warming treatments induced diverse ER increase rates during the growing season (Figs. 4A–4E). CK and W3 had the lowest (0.64 µmol m^−2^ s^−1^ per 1 °C temperature increase, Fig. 4A) and highest (1.38 µmol m^−2^ s^−1^ per 1 °C temperature increase, Fig. 4D) rate of increase, respectively. Other warming treatments had a similar rate of increase of approximately 1.0µmol m^−2^ s^−1^ per 1 °C temperature increase (Figs. 4B, 4C, 4E).

ER responded non-linearly to gradient warming (Fig. 3B). As temperatures increased, ER showed a convex parabolic curve with its peak value appearing between 1,500 W and 2,000 W warming, whereas the highest rate increase occurred at 1,500 W warming.

With the combined effects of measuring dates and the warming treatments, ER increased linearly with the increasing temperature (Fig. 4F). A 1 °C temperature increase resulted in an ER increase of 0.92 µmol m^−2^ s^−1^ with an R^2^ of 0.63.

### Effects of warming on GPP

The measuring dates, warming treatments, and their interactions also significantly affected GPP (Table 1, *p* < 0.01).

**Figure 5:**
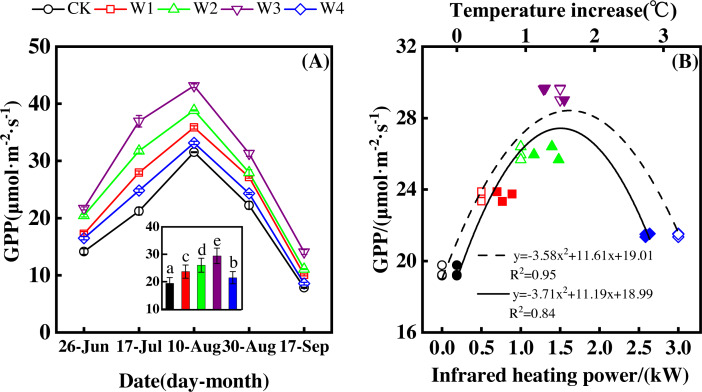
Responses of gross primary productivity (GPP) to measuring dates (A) and warming treatments (B) during the growing season. The warming treatments were CK (no warming), W1 (500 W warming), W2 (1,000 W warming), W3 (1,500 W warming), and W4 (3,000 W warming). The hollow symbols and dotted line were the responses to temperature, while the solid symbols and line were the responses to the warming treatments.

Measuring dates significantly affected GPP, resulting in a convex parabolic curve of GPP during the growing season (Fig. 5A). From late June to early August, GPP monotonically increased and peaked (31.52–43.14 µmol m^−2^ s^−1^) in early August. GPP began to decrease after early August and reached its lowest values (7.82–14.06 µmol m^−2^ s^−1^) at the end of September.

GPP also significantly differed among treatments (Fig. 5A, *p* < 0.05). All warming treatments took a significantly higher GPP than the control (CK). In addition, GPP differed significantly among warming treatments. The highest GPP corresponded to a GPP increase of 10.05 µmol m^−2^ s^−1^ at 1,500 W warming (W3), which was associated with a temperature increase of 1.37 °C. 500 W warming (W1) and 1,000 W warming (W2) led to a GPP increase of 4.27 µmol m^−2^ s^−1^, and 6.63 µmol m^−2^ s^−1^, respectively. 3,000 W warming (W4) only increased GPP 2.07 µmol m^−2^ s^−1^.

**Figure 6:**
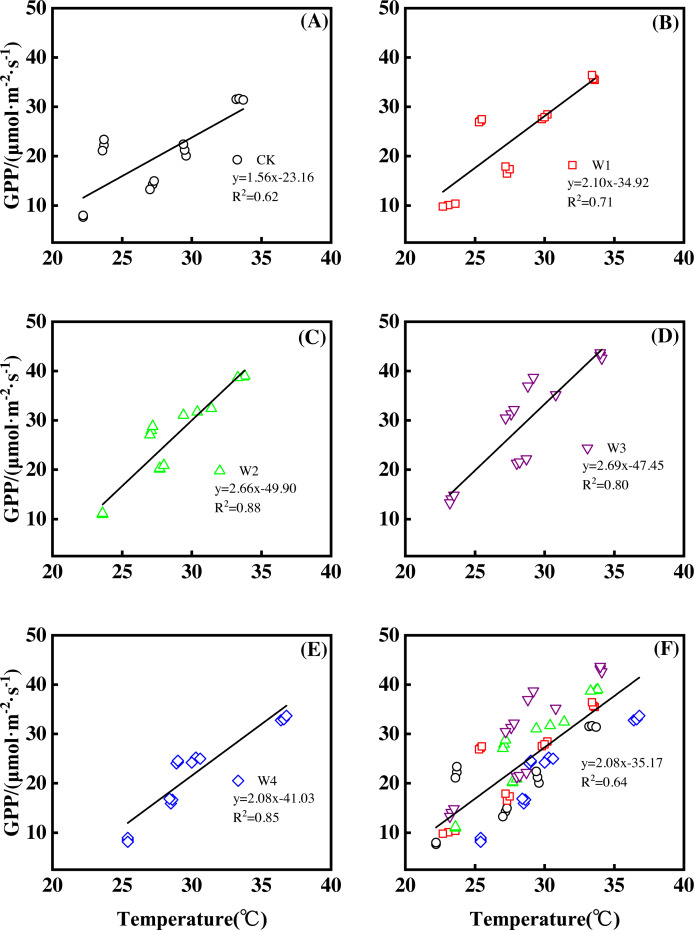
Responses of gross primary productivity (GPP) to temperature under different warming treatments (A–E) and under the combined effects (f) during the growing season. (A–E) were the responses of GPP to temperature under CK (no warming, a), W1 (500 W warming, (B), W2 (1,000 W warming, (C), W3 (1,500 W warming, (D), and W4 (3,000 W warming, (E), respectively, while f was the responses of GPP to temperature under the combined effects of measuring dates and warming treatments.

Furthermore, different warming treatments resulted in diverse GPP increase rates (Figs. 6A–6E) during the growing season. CK had the lowest rate of increase (1.56µmol m^−2^ s^−1^ per 1 °C temperature increase, Fig. 6A), whereas W2 and W3 had the highest rate of increase around 2.6 µmol m^−2^ s^−1^ per 1 °C temperature increase (Figs. 6C–6D). Other warming treatments had a similar rate of increase around 2.0 µmol m^−2^ s^−1^ per 1 °C temperature increase (Figs. 6B, 6E).

GPP responded non-linearly to the gradient warming (Fig. 5B). As temperatures increased, GPP showed a convex parabolic curve, with its peak value occurring between 1,500 W and 2,000 W warming, whereas the highest rate of increase occurred at 1,500 W warming.

GPP linearly increased with the increasing temperature when the combined effects of measuring dates and warming treatments were considered (Fig. 6F). A 1 °C temperature increase resulted in a GPP increase of 2.08 µmol m^−2^ s^−1^ with an R^2^ of 0.64.

### Relationships between NEP, GPP, and ER

**Figure 7:**
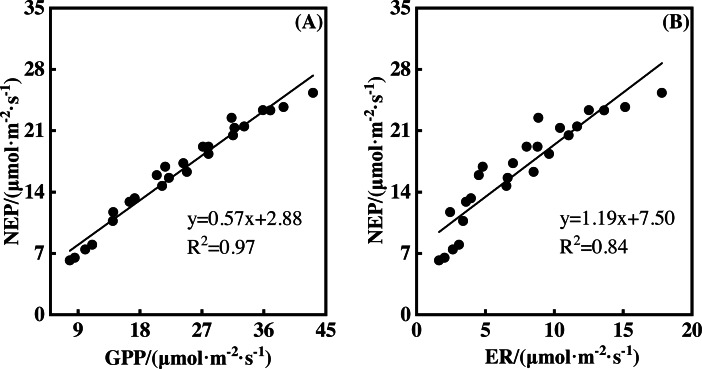
The relationships between gross primary productivity (GPP) and net ecosystem productivity (NEP), ecosystem respiration (ER) and NEP.

NEP showed a significantly positive relationship with GPP during the measurement period. This was also true for the relationship between NEP and ER (Fig. 7, *p* <0.01). A 1 µmol m^−2^ s^−1^ GPP increase led to an NEP increase of 0.57 µmol m^−2^ s^−1^, with an R^2^ of 0.97. However, a 1 µmol m^−2^ s^−1^ ER increase resulted in an NEP increase of 1.19 µmol m^−2^ s^−1^, with an R^2^ of 0.84. Therefore, GPP showed a closer relationship with NEP than ER.

## Discussion

In this study, we found that measuring dates, warming treatments, and their interactions all had significant impacts on NEP. All warming treatments (W1, W2, W3, and W4) significantly increased NEP. However, the highest NEP appeared at the W3 treatment, as did ER and GPP, revealing a non-linear response of NEP to gradient warming. In addition, GPP and ER were both positively related to the variation of NEP.

Our findings confirmed the promotion effect of warming (Ganjurjav et al., 2015; Welker et al., 2004). For example, Welker et al. (2004) found an increasing NEP after warming by conducting a warming experiment in a High Arctic tundra, which increased the air temperature approximately 1–1.5 °C. Ganjurjav et al. (2015) found that warming in an alpine meadow on the central Qinghai-Tibetan Plateau prompted NEP. Though some previous works found that NEP decreased with the increasing temperature (Jiang et al., 2012; Wang et al., 2019a), the decreasing NEP in previous works may have been the result of drought. During drought, the increasing temperature may stimulate decomposition and ER but may contribute little to GPP as water, which results in an NEP decrease. Few attempts have been made to use manipulating experiments to analyze how gradient warming affects NEP and models are typically used to conduct such analysis (Barron-Gafford et al., 2012; Yi et al., 2019). Model simulations suggest that warming under 2 °C would increase the net carbon uptake (Wang et al., 2019b; Yi et al., 2019), whereas excessive warming would flatten the increasing carbon uptake rate (Barron-Gafford et al., 2012; Huang et al., 2011), which supports our results. NEP did increase with the rising temperature but responded non-linearly to different warming magnitudes.

The non-linear responses of NEP to gradient warming resulted from the difference between GPP and ER (Chapin et al., 2006). The non-linear responses of GPP (Fig. 5B) and ER (Fig. 3B) to gradient warming resulted in a non-linear response of NEP. GPP provided the substrate of plant autotrophic respiration (AR) (Chapin et al., 2006), which was the main component of ER in a flood ecosystem and would govern the responses of ER. Therefore, GPP contributed more to the variation of NEP, indicated by the close linear relationship between GPP and NEP (Fig. 7).

The effect of temperature on GPP may be due to a stoma restriction and a non-stoma restriction. The varying temperature may alter stoma conductance, leading to a change in the amount of CO_2_ entering the intracellular spaces and altering the photosynthetic rate and GPP (Berry & Bjorkman, 1980). Temperature changes may also affect GPP by altering chloroplast structures, which affected the enzyme activity responsible for catalysing the carbon-fixation reaction. The increasing temperature may promote stoma conductance and exert a non-linear effect on enzyme activity. A moderate increase of temperature may activate the enzyme activity, prompting GPP, whereas excessive warming would cause the protein to degenerate and inhibit the increasing magnitude of GPP. Therefore, GPP responded non-linearly to increasing temperatures.

The non-linear responses of ER to different warming magnitudes may be explained by the metabolism theory. According to the metabolism theory, ER may increase with the increasing temperature and substrate amount (Enquist et al., 2003; Yvon-Durocher et al., 2012). Temperatures increased linearly with the increasing warming magnitudes (Fig. 1B, 3B and 5B), leading to a linear ER increase when the substrate amount varied little (Enquist et al., 2003; Yvon-Durocher et al., 2012). However, GPP, the substrate of AR, responded non-linearly to the increasing warming magnitudes, leading to a non-linear AR increase (Yvon-Durocher et al., 2012). Additionally, the continuously flooded environment may lead to poorer decomposition of soil organic matter and little change in heterotrophic respiration (HR). Therefore, ER, the sum of AR and HR, was encouraged by warming but it responded non-linearly to different warming magnitudes.

Warming increases of GPP and ER resulted in the NEP increase. Warming increased GPP and ER with a rate of 2.07 to 10.05 µmol m^−2^ s^−1^ (Fig. 5A) and 0.91 to 4.04 µmol m^−2^ s^−1^ (Fig. 3A), respectively, leading to the NEP increase of 1.16 to 6.01 µmol m^−2^ s^−1^ (Fig. 1A). In addition, the portion of ER to GPP was usually consistent over time (Baldocchi, Sturtevant & Contributors, 2015; Chen et al., 2015; Collalti & Prentice, 2019), indicating that similar portion of GPP was released by ER. Therefore, NEP increased with warming but responded non-linearly to gradient warming (Fig. 1B), resulting from the non-linear responses of GPP (Fig. 5B) and ER (Fig. 3B).

This study only reflected the response of NEP in a paddy field in Northeast China. The responses of other ecosystem types to gradient warming should be investigated in the future. In addition, a 1 °C temperature increase resulted in a variety of increasing rates of NEP and its components among warming treatments (Figs. 2, 4 and 6), which also needs further analysis.

## Conclusions

We investigated the responses of net ecosystem productivity to different warming magnitudes using the static chamber-infrared gas analyser method in a manipulating experiment in a paddy field in Northeast China. Results showed that warming significantly promoted NEP and its components. However, NEP and its components responded non-linearly to the increasing temperatures. The responses of ecosystem respiration and NEP to warming magnitudes resulted from those of gross primary productivity. Therefore, GPP responded non-linearly to gradient warming resulted in a non-linear response of ER and NEP. The non-linear effects of warming should be taken into account in the future.

##  Supplemental Information

10.7717/peerj.9327/supp-1 Data S1 NEP data in growing season rice field ecosystem under gradient warming treatment

10.7717/peerj.9327/supp-2 Data S2 GPP data in growing season rice field ecosystem under gradient warming treatment

10.7717/peerj.9327/supp-3 Data S3 ER data in growing season rice field ecosystem under gradient warming treatment

10.7717/peerj.9327/supp-4 Data S4 Temperature data in growing season rice field ecosystem under gradient warming treatment

## Additional Information and Declarations

### Data Availability

The following information was supplied regarding data availability: The raw measurements of NEP, ER and GPP and the raw temperature data are available as Supplemental Files.

## References

[ref-1] Baldocchi D, Sturtevant C, Contributors F (2015). Does day and night sampling reduce spurious correlation between canopy photosynthesis and ecosystem respiration?. Agricultural and Forest Meteorology.

[ref-2] Barron-Gafford GA, Scott RL, Jenerette GD, Hamerlynck EP, Huxman TE (2012). Temperature and precipitation controls over leaf- and ecosystem-level CO2 flux along a woody plant encroachment gradient. Global Change Biology.

[ref-3] Berry J, Bjorkman O (1980). Photosynthetic response and adaptation to temperature in higher plants. Annual Review of Plant Physiology.

[ref-4] Blumenthal DM, Kray JA, Ortmans W, Ziska LH, Pendall E (2016). Cheatgrass is favored by warming but not CO_2_ enrichment in a semi-arid grassland. Global Change Biology.

[ref-5] Chapin FS, Woodwell GM, Randerson JT, Rastetter EB, Lovett GM, Baldocchi DD, Clark DA, Harmon ME, Schimel DS, Valentini R, Wirth C, Aber JD, Cole JJ, Goulden ML, Harden JW, Heimann M, Howarth RW, Matson PA, McGuire AD, Melillo JM, Mooney HA, Neff JC, Houghton RA, Pace ML, Ryan MG, Running SW, Sala OE, Schlesinger WH, Schulze ED (2006). Reconciling carbon-cycle concepts, terminology, and methods. Ecosystems.

[ref-6] Chen Z, Yu GR, Zhu XJ, Wang QF, Niu SL, Hu ZM (2015). Covariation between gross primary production and ecosystem respiration across space and the underlying mechanisms: a global synthesis. Agricultural and Forest Meteorology.

[ref-7] Collalti A, Prentice IC (2019). Is NPP proportional to GPP? Waring’s hypothesis 20 years on. Tree Physiology.

[ref-8] Dong WJ, Deng AX, Zhang B, Tian YL, Chen J, Yang F, Zhang WJ (2011). Experimental study on the effects of different day and night warming on single-season rice. Acta Ecologica Sinica.

[ref-9] Enquist BJ, Economo EP, Huxman TE, Allen AP, Ignace DD, Gillooly JF (2003). Scaling metabolism from organisms to ecosystems. Nature.

[ref-10] Fang JY, Zhu JL, Wang SP, Yue C, Shen HH (2011). Global warming, human-induced carbon emissions, and their uncertainties. Science China Earth Sciences.

[ref-11] Fu BJ, Niu D, Zhao SD (2005). Global change and terrestrial ecosystems: review and Prospect. Advances in Earth Sciences.

[ref-12] Fu Z, Niu SL, Dukes JS (2015). What have we learned from global change manipulative experiments in China? A meta-analysis. Scientific Reports.

[ref-13] Ganjurjav H, Gao QZ, Zhang WN, Liang Y, Li YW, Cao XJ, Wan YF, Li Y, Danjiu LB (2015). Effects of warming on CO_2_ fluxes in an alpine meadow ecosystem on the Central Qinghai-Tibetan Plateau. PLOS ONE.

[ref-14] Gao QZ, Duan MJ, Wan YF, Li YE, Guo YQ, Jiang-Cun WZ (2010). Assessment of ecological and environmental sensitivity in northern Tibet. Acta Ecologica Sinica.

[ref-15] Huang S, Arain MA, Arora VK, Yuan FM, Brodeur J, Peich IM (2011). Analysis of nitrogen controls on carbon and water exchanges in a conifer forest using the CLASS-CTEM^N+^ model. Ecological Modelling.

[ref-16] Jiang L, Guo R, Zhu TC, Niu XD, Guo JX, Sun W (2012). Water- and plant-mediated responses of ecosystem carbon fluxes to warming and nitrogen addition on the Songnen grassland in northeast China. PLOS ONE.

[ref-17] Jones MH, Fahnestock JT, Walker DA, Walker MD, Welker JM (1998). Carbon dioxide fluxes in moist and dry arctic tundra during the snow-free season: responses to increases in summer temperature and winter snow accumulation. Arctic and Alpine Research.

[ref-18] Liu XD, Chen BD (2000). Climatic warming in the Tibetan plateau during recent decades. International Journal of limatology.

[ref-19] Lupascu M, Welker JM, Seibt U, Maseyk K, Xu X, Czimczik CI (2013). High Arctic wetting reduces permafrost carbon feedbacks to climate warming. Nature Climate Change.

[ref-20] Ma ZY, Liu HY, Mi ZR, Zhang ZH, Wang YH, Xu W, Jiang L, He J-S (2017). Climate warming reduces the temporal stability of plant community biomass production. Nature Communications.

[ref-21] Mu CC, Zhang TJ, Zhao Q, Su H, Wang SF, Cao B, Peng XQ, Wu QB, Wu XD (2017). Permafrost affects carbon exchange and its response to experimental warming on the northern Qinghai-Tibetan Plateau. Agricultural and Forest Meteorology.

[ref-22] Nijs I, Ferris R, Blum H, Hendrey G, Impens I (1997). Stomatal regulation in a changing climate: a field study using Free Air Temperature Increase (FATI) and Free Air CO_2_ Enrichment (FACE). Plant, Cell & Environment.

[ref-23] Niu SL, Sherry RA, Zhou XH, Luo YQ (2013). Ecosystem carbon fluxes in response to warming and clipping in a tallgrass Prairie. Ecosystems.

[ref-24] Oberbauer SF, Tweedie CE, Welker JM, Fahnestock JT, Henry GHR, Webber PJ, Hollister RD, Walker MD, Kuchy A, Elmore E, Starr G (2007). Tundra Co2fluxes in Response To Experimental Warming across Latitudinal and Moisture Gradients. Ecological Monographs.

[ref-25] Pendall E, Heisler-White JL, Williams DG, Dijkstra FA, Carrillo Y, Morgan JA, Lecain DR (2013). Warming reduces carbon losses from grassland exposed to elevated atmospheric carbon dioxide. PLOS ONE.

[ref-26] Sharp ED, Sullivan PF, Steltzer H, Csank AZ, Welker JM (2013). Complex carbon cycle responses to multi-level warming and supplemental summer rain in the high Arctic. Global Change Biology.

[ref-27] Tokarska KB, Gillett NP (2018). Cumulative carbon emissions budgets consistent with 1.5 °C global warming. Nature Climate Change.

[ref-28] Wang ZQ, Chang JF, Peng SS, Piao SL, Ciais P, Betts R (2019b). Changes in productivity and carbon storage of grasslands in China under future global warming scenarios of 1.5 degrees C and 2 degrees C. Journal of Plant Ecology.

[ref-29] Wang N, Quesada B, Xia L, Butterbach-Bahl K, Goodale CL, Kiese R (2019a). Effects of climate warming on carbon fluxes in grasslands—a global meta-analysis. Global Change Biology.

[ref-30] Welker JM, Fahnestock JT, Henry GHR, O’Dea KW, Chimner RA (2004). CO_2_ exchange in three Canadian High Arctic ecosystems: response to long-term experimental warming. Global Change Biology.

[ref-31] WMO (2019). WMO Statement on the status of the global climate in 2018.

[ref-32] Wolkovich EM, Cook BI, Allen JM, Crimmins TM, Betancourt JL, Travers SE, Pau S, Regetz J, Davies TJ, Kraft NJB (2012). Warming experiments underpredict plant phenological responses to climate change. Nature.

[ref-33] Xu X, Shi Z, Li DJ, Zhou XH, Sherry RA, Luo YQ (2015). Plant community structure regulates responses of prairie soil respiration to decadal experimental warming. Global Change Biology.

[ref-34] Yi SH, Xiang B, Meng BP, Wu XD, Ding YJ (2019). Modeling the carbon dynamics of alpine grassland in the Qinghai-Tibetan Plateau under scenarios of 1.5 and 2 degrees C global warming. Advances in Climate Change Research.

[ref-35] Yvon-Durocher G, Caffrey JM, Cescatti A, Dossena M, Giorgio PD, Gasol JM, Montoya JM, Pumpanen J, Staehr PA, Trimmer M, Woodward G, Allen AP (2012). Reconciling the temperature dependence of respiration across timescales and ecosystem types. Nature.

[ref-36] Yvon-Durocher G, Jones JI, Trimmer M, Woodward G, Montoya JM (2010). Warming alters the metabolic balance of ecosystems. Philosophical Transactions of the Royal Society B-Biological Sciences.

